# Experimental Evaluation of Nonlinear Parameters in Fatigue Crack Growth Using Digital Image Correlation

**DOI:** 10.3390/ma18225110

**Published:** 2025-11-10

**Authors:** Giancarlo L. Gómez Gonzales, Francisco A. Díaz

**Affiliations:** Department of Mechanical and Mining Engineering, University of Jaén, 23071 Jaén, Spain

**Keywords:** fatigue crack propagation, crack-tip plasticity, high-resolution digital image correlation

## Abstract

This study presents an experimental methodology for characterizing the crack-tip region using high-resolution Digital Image Correlation (DIC). The approach utilizes a stereoscopic microscope setup combined with 3D-DIC analysis to enable precise measurements within the small-scale region surrounding the crack tip. Two nonlinear parameters are evaluated: the plastic component of the crack-tip opening displacement (CTOD_p_) and the cyclic plastic zone size. The investigation was conducted on disk-shaped compact tension specimens made of AISI 1020 steel under constant-ΔK fatigue testing. The results demonstrate a strong correlation between these nonlinear parameters and fatigue crack propagation, which was maintained stable, validating the proposed methodology. Furthermore, the relevance of crack-tip plasticity in fatigue crack propagation is verified under the tested conditions, highlighting its utility for fatigue life assessment under complex loading scenarios.

## 1. Introduction

The stress intensity factor range (ΔK) is a fundamental linear elastic parameter proposed by Paris [1] in the 1960s for the assessment of fatigue crack propagation. This parameter relates the crack growth rate per cycle (da/dN) to ΔK in a log–log linear relationship. However, da/dN-ΔK curves have limitations, such as sensitivity to stress ratio (R). Furthermore, it shows limitations to incorporate load history effects and also in predicting the behavior of short cracks. It is known that the ΔK parameter is based on linear elastic fracture mechanics and becomes questionable when applied in conditions of significant plasticity. In 1970, Elber proposed one of the most commonly adopted models to explain fatigue crack growth behavior: the plasticity-induced crack closure [2]. This model considers that the residual plastic deformation left in the crack wake induces premature closure of the crack flanks, even when the minimum load of the cycle is not reached [3]. However, despite its widespread use, the validity of the plasticity-induced crack closure continues to be questioned under certain conditions [4,5,6,7,8]. Other approaches include the T-stress parameter [9], complex models like Christopher-James–Patterson (CJP) [10], and methods focusing on K_max_ and ΔK that emphasize the mechanisms occurring at the crack front [11,12]. On the other hand, alternative approaches based on elastic–plastic fracture mechanics (EPFM) are gaining relevance due to their improved ability to characterize the local plasticity at the crack tip, which is primarily associated with fatigue damage.

Various studies have focused on nonlinear parameters, notably the cyclic plastic strain range, the size of the cyclic plastic zone, the plastic component of the crack-tip opening displacement (CTOD_p_), and plastic energy dissipation. A key attribute of these parameters is their ability to directly quantify the plasticity at the crack tip, showing consistent correlations with crack growth rates across a wide range of loading scenarios [13,14]. Consequently, they are increasingly recognized as more reliable physical parameters governing crack propagation.

In 1966, Burdekin and Stone [15] introduce the use of crack-tip opening displacement (CTOD) in fracture mechanics for cases where linear elastic fracture mechanics (LEFM) fails due to yielding conditions. A theoretical model was also developed to describe the relationship between stress, strain, crack length, and CTOD. Based on this study, CTOD has been widely adopted as a characterizing parameter. Donahue et al. [16] demonstrated that propagation rates across various materials could be effectively correlated using crack opening displacement and two material constants, including the threshold stress intensity factor. Shahani et al. [17] compared multiple fracture mechanics parameters, concluding that the j-integral and the CTOD provided better correlations with fatigue crack growth rates. More recently, Antunes et al. [18] showed through numerical studies that the plastic component of the CTOD has a linear relationship with fatigue crack growth rates. This relation naturally incorporates phenomena such as crack closure, crack tip blunting, residual stress, and the fatigue threshold. Subsequent experimental work, including fatigue crack growth tests on aluminum and titanium specimens by Antunes et al. [19] and Vasco et al. [20,21], confirmed that the da/dN–CTOD_p_ relationship remains independent of the stress ratio.

On the other hand, among the damage mechanisms operating at the crack tip, cyclic plastic deformation is established as the dominant factor by many researchers [22,23]. The cyclic plastic zone is a region within the monotonic plastic zone where the material undergoes plastic deformation during both the loading and unloading phases of a complete loading cycle. During the loading phase, the material yields in tension, while during unloading to the minimum load, it yields in compression. For this reason, it is also referred to in the literature as the reversed plastic zone (rpz). This contrasts with the monotonic plastic zone, where plastic deformation occurs primarily during the loading phase.

Several studies [24,25,26,27] suggested that cyclic plastic strain is a key mechanism in fatigue crack growth under cyclic loading based on the observed significant correlation between cyclic plastic zone size and crack growth rates. Fatigue crack growth is closely related to the cyclic plastic zone, where cyclic plastic deformation occurs and fatigue damage is most severe. Larger cyclic plastic zone sizes lead to greater energy loss during crack growth, while smaller sizes require less energy.

This has led to a growing emphasis on experimental and numerical methods with the capacity of capturing localized plasticity fields, particularly under complex loading. Consequently, the application of nonlinear fracture parameters not only enhances the predictive capability of fatigue models but also provides a deeper, physically grounded understanding of the micromechanical processes governing fatigue crack propagation.

In experimental mechanics, characterizing the region near the crack tip is particularly challenging due to its small size and high strain gradient. In this context, the DIC technique has gained increasing popularity in fatigue crack propagation studies, as it enables highly accurate measurements across various scales through the appropriate selection of optical systems and imaging devices. However, the experimental procedure requires careful surface preparation, optimal speckle pattern application, and precise calibration of the optical setup to ensure reliable and repeatable results. Although few studies are available in the literature [28,29,30,31], all of them highlight the importance of using an appropriate methodology to enhance the accuracy of DIC measurements.

This paper aims to analyze two nonlinear parameters: the plastic crack-tip opening displacement and the cyclic plastic zone size. To achieve this, a stereoscopic microscope setup combined with 3D Digital Image Correlation (3D-DIC) analysis was employed. A dedicated methodology was developed to produce an appropriate speckle pattern using black toner powder, ensuring accurate and reliable measurements. Both parameters were evaluated in a pre-cracked specimen subjected to a constant-ΔK test, which differs from previous studies that employed constant-ΔP conditions. In a constant-ΔP test, the applied load range remains fixed throughout the experiment, leading to a continuous increase in the stress intensity factor (ΔK) as the crack grows. In contrast, in a constant-ΔK test, the load is progressively reduced during crack propagation to maintain a nearly constant stress intensity range. This approach enables a more controlled assessment of crack growth behavior and fracture parameters, as it isolates the effect of ΔK from the influence of changing load conditions. The use of the constant-ΔK approach is justified, as it allows a more direct assessment of crack-shielding mechanisms, such as plasticity-induced crack closure, by eliminating variations in crack-tip driving force caused by increasing ΔK. Under constant ΔK, it is expected that the effective force or forces governing crack propagation should also remain unchanged. The results obtained for the plastic crack-tip opening displacement and the cyclic plastic zone size from the steel specimens are consistent with this premise, thereby validating the methodology for their measurement and demonstrating their utility for fatigue life assessments.

Additionally, the use of 3D-DIC analysis offers greater reliability due to the high-resolution measurements, capturing features such as surface roughening or depressions resulting from large plasticity near the crack tip, which remain undetectable with conventional 2D-DIC analysis.

## 2. Materials and Methods

**Figure 1:**
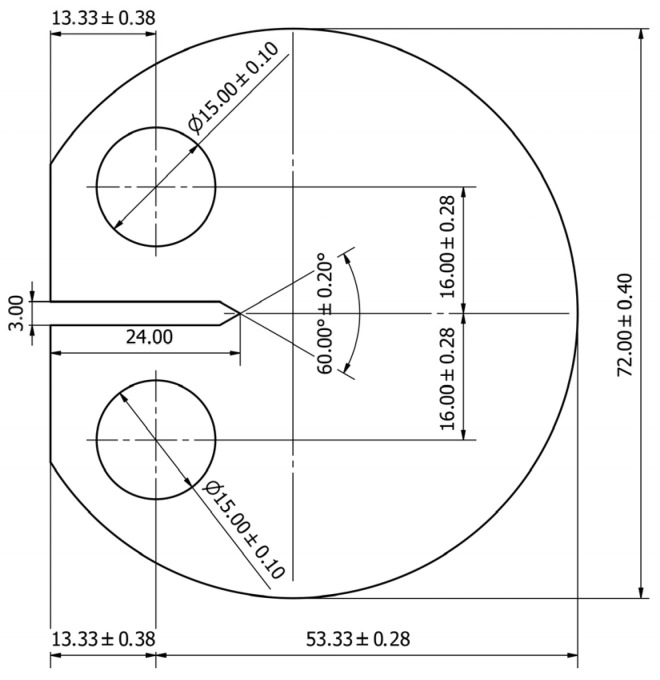
Shape and dimensions of the DCT specimen employed in this study [34].

The experimental specimens were machined from a 76 mm-diameter AISI 1020 steel bar into disk-shaped compact tension (DCT) samples with a thickness of 5 mm, following the geometry detailed in ASTM E647 [32]. The complete dimensions of the samples (all in millimeters) are shown in Figure 1. The material’s mechanical behavior, including monotonic and cyclic properties, is summarized in Table 1. The chemical composition of the AISI 1020 steel used in the experiments is presented in Table 2.

**Table 1:** Material properties of AISI 1020 steel considered [33,34].

Young’s Modulus (E)	206 GPa
Tensile yield strength (σ_y_)	285 MPa
Cyclic yield strength (σ_cy_)	270 MPa
Cyclic strain hardening exponent (h′)	0.18
Cyclic strength coefficient (H′)	941 MPa

**Table 2:** Chemical composition for commercially pure AISI 1020 steel.

Element	C	Mn	Si	Cu	Cr	Ni	S	P
Content (wt.%)	0.20	0.55	0.12	0.18	0.07	0.05	0.022	0.019

**Figure 2:**
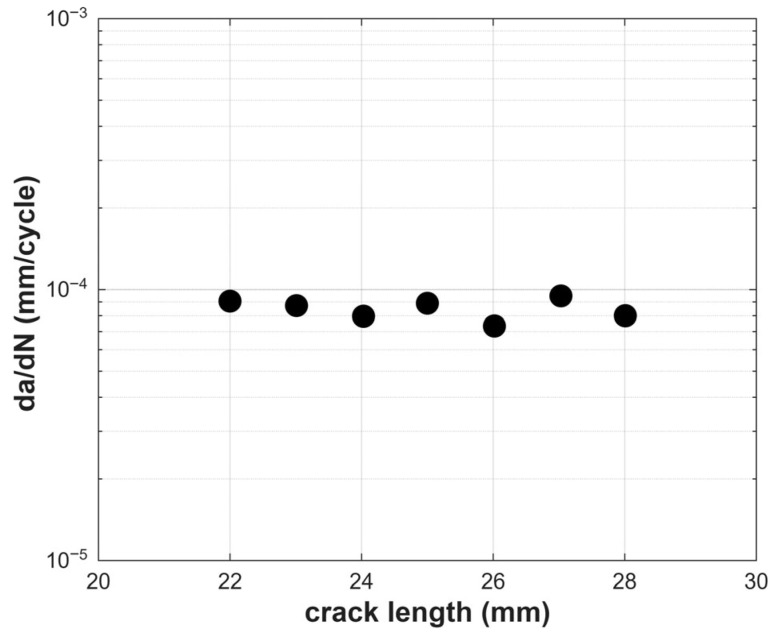
Fatigue crack propagation rates da/dN measured along the experiment.

The fatigue test in Mode I loading conditions was performed in accordance with the ASTM E647 standard. The experiments were carried out on a servo-hydraulic Instron universal testing machine equipped with a 100 kN load cell. The test involved fatigue crack propagation under constant stress intensity factor range, ΔK = 30 MPa√m, and a load ratio of R = 0.1. The objective of a constant-ΔK test is to maintain a fixed range of ΔK throughout the crack growth process, regardless of the crack length. To achieve this, the applied load is progressively decreased as the crack advances in order to compensate for the reduction in specimen stiffness. This approach allows for a stable crack growth rate and facilitates the investigation of fatigue behavior under constant driving force conditions. Figure 2 presents the fatigue crack propagation rates in terms of the crack length increment per cycle, da/dN. It is evident that under a consistently imposed ΔK, da/dN remains nearly constant, with variations limited to the noise level associated with the required load adjustments to maintain constant-ΔK conditions.

**Figure 3:**
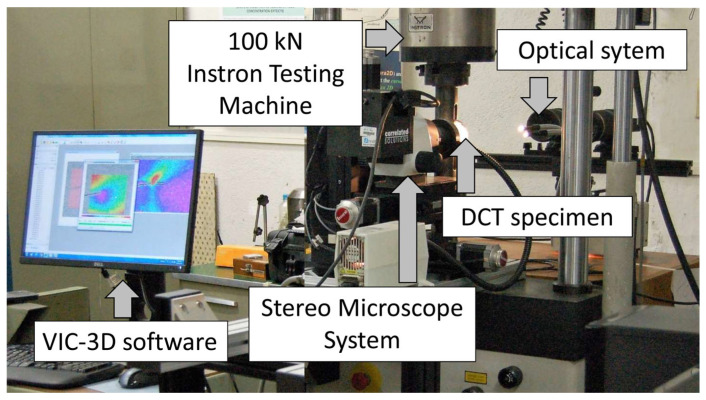
Stereo microscope system lens positioned in front of the region of interest.

For this analysis, a stereo microscope system from Correlated Solutions (Columbia, SC, USA) was used, consisting of two 5 MP Point Grey cameras coupled with an Olympus SZX16 microscope (Olympus, Tokyo, Japan), as shown in Figure 3. The system includes distortion correction algorithm to ensure accurate measurements at high magnification. Prior to the test, the stereo system was carefully calibrated to ensure precise three-dimensional measurement of out-of-plane displacements. Using this optical setup, a 3 × 3.6 mm region ahead of the crack tip was analyzed with a spatial resolution of approximately 1.6 μm/pixel. The fine speckle pattern necessary for high-quality DIC at this magnification was achieved by applying black toner powder over a white-painted surface.

After the crack reached approximately 20 mm of propagation, images were captured during cyclic loading at various crack lengths. For image acquisition, the test frequency was reduced from its nominal value to 0.01 Hz, allowing the capture of at least 100 consecutive stereo images during each monitored cycle. All images were processed using the VIC-3D software (version 8.0.0) from Correlated Solutions, which performs full-field 3D-DIC analysis. For the DIC analysis, a subset size (SS) of 41 pixels was selected to ensure that each subset could be uniquely distinguished from the others. A step size (ST) of 11 pixels and a strain window (SW) of 19 data points were used. The correlation algorithm employed was the normalized sum-of-squared-differences, combined with an optimized 8-tap interpolation to compensate for subset smoothing by using a larger subset. All DIC parameters were selected in accordance with the recommendations of the Good Practices Guide for DIC [35].

To evaluate the accuracy of the selected DIC parameters, an assessment of the measurement resolution was performed. For practical purposes, the resolution of the DIC measurements is defined as the standard deviation of the displacement or strain field obtained under conditions where the true value is expected to be zero. To determine this, two images of the specimen taken in the unloaded condition were analyzed. This analysis represents the experimental uncertainty of the displacement or strain measurements and defines the smallest detectable variation that can be distinguished from noise. In the present study, the displacement resolution was determined to be approximately 8.67 × 10^−2^ µm, while the corresponding strain resolution was around 80 µε.

### 2.1. Experimental Determination of the Plastic CTOD

**Figure 4:**
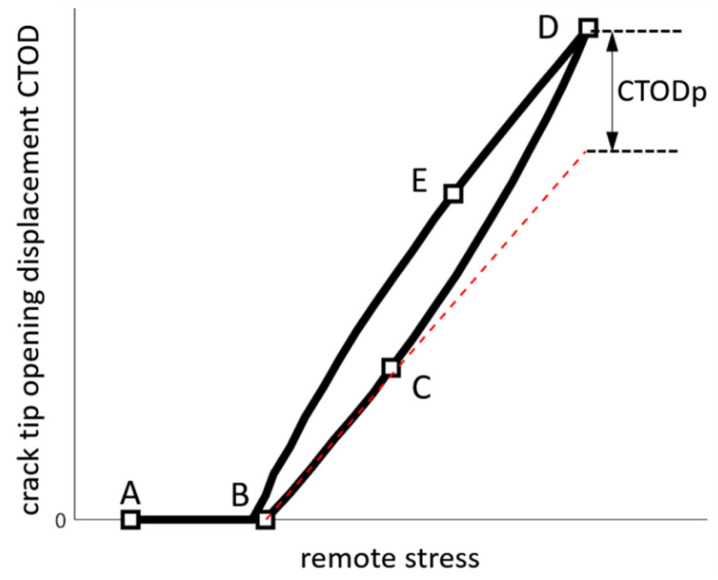
CTOD values over a complete loading cycle and determination of CTOD_p_.

The plastic CTOD parameter is directly obtained from a CTOD curve, which is a fracture mechanics measure of the displacement between the two crack faces when the crack is open. Figure 4 shows a typical CTOD curve obtained from a cracked specimen for a ductile material under cyclic loading. The segment AB, where the CTOD values remain at zero, indicates that the crack is closed even when load is being applied. This behavior is typical in ductile materials and corresponds to the crack closure phenomenon. As the load increases, the crack opens following elastic behavior, as shown in segment BC. Beyond this point, the curve changes its slope, indicating plastic deformation at the crack tip region as the load approaches its maximum value. During the unloading phase, the segment DE runs parallel to segment BC, exhibiting a similar behavior. Therefore, the slope of the unloading segment DE is useful for identifying the material’s elastic response at BC. This involves extrapolating the elastic segment BC, which represents the material’s elastic response, and removing the elastic contribution from the total CTOD measurement to obtain the plastic CTOD.

The previously described methodology was implemented in a custom MATLAB (version R2021a) program, which uses the relative displacement between the opposite crack faces as input. To determine this displacement, several pairs of symmetrical points distributed along the crack faces are carefully identified on the vertical displacement map obtained from the DIC analysis. The total CTOD is evaluated using the formula CTOD = *v*_upper_ − *v*_lower_, where *v* represents the local vertical displacement. CTOD values are extracted during both the loading and unloading phases. The MATLAB code processes this data to determine the maximum value of the plastic CTOD. This automated approach enhances the consistency and repeatability of the analysis while also improving the overall accuracy of the measurements.

### 2.2. Experimental Determination of the Cyclic Plastic Zone

The cyclic plastic zone is a region near the crack tip where the material undergoes plastic deformation in the opposite direction during the unloading segment in a fatigue cycle. Therefore, to evaluate if plastic strain develops during the unloading path, the cyclic yield stress (σ_cy_) is used. This parameter quantifies the stress level point in the ∆σ∆ε loop at which the material begins to plastically deform during repeated loading. The cyclic yield stress is typically lower than the monotonic yield stress due to strain hardening, microstructural evolution, and cyclic softening or hardening.

**Figure 5:**
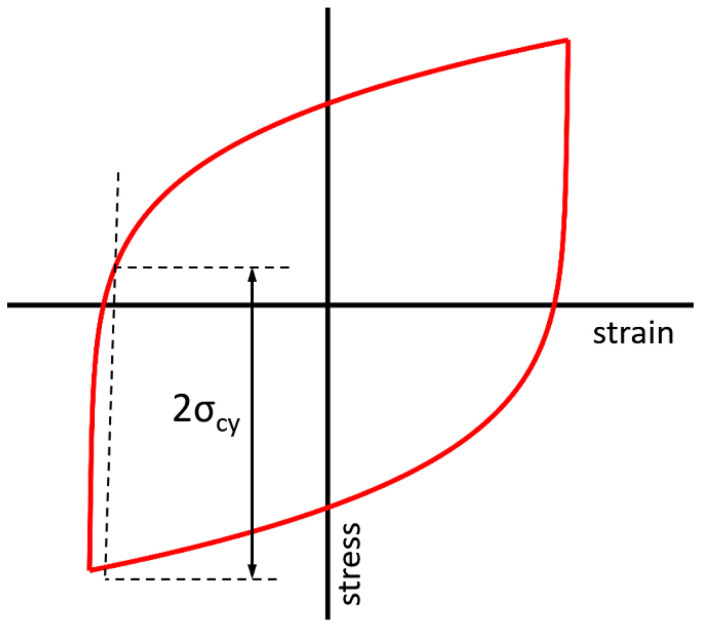
Procedure to determine cyclic yield stress from a cyclic stress–strain loop.

In the procedure used to estimate the cyclic yield stress, the value 2·σ_cy_ is used as a reference criterion, based on analysis of the loading and unloading segments of the stress–strain curve (see Figure 5). Therefore, a region ahead of the crack tip is considered to be within the cyclic plastic zone if the stress range (Δσ) experienced during unloading exceeds 2·σ_cy_.

To identify this region experimentally, vertical strain maps obtained via DIC analysis are investigated. For each measurement point ahead of the crack tip, the corresponding cyclic strain range (Δ*ε*) is calculated and then converted to Δ*σ* using the Ramberg–Osgood relationship:

(1) $$\frac{\Delta \varepsilon }{2}=\frac{\Delta \sigma }{2E}+{\left(\frac{\Delta \sigma }{2H^{\prime }}\right)}^{\frac{1}{h^{\prime }}}$$

where *E* is Young’s Modulus, *H*′ is the cyclic strength coefficient, and *h*′ is the cyclic strain hardening exponent.

Therefore, the cyclic plastic zone develops at all points where the stress range exceeds 2·*σ*_cy_. To efficiently process the DIC measurements and accurately determine both the size and detailed shape of this cyclic plastic zone, a specialized custom MATLAB program was specifically developed and implemented for automated analysis.

## 3. Results

**Figure 6:**
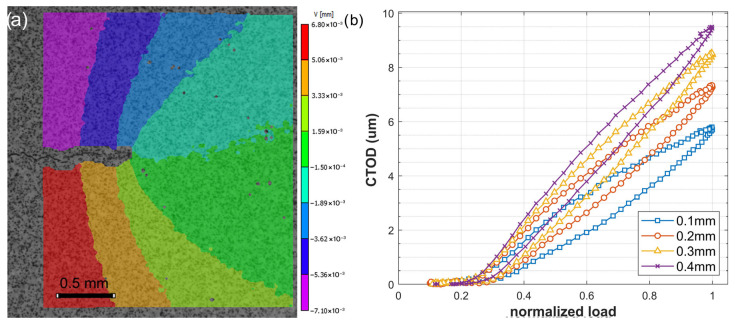
(**a**) Vertical displacement map obtained via DIC at a crack length of 22 mm. (**b**) CTOD profiles measured at various positions behind the crack tip.

The first step involves obtaining the vertical displacement evolution under cyclic loading. Figure 6a shows the vertical displacement field at a crack length of 22 mm under maximum loading conditions, where the crack is fully open. It can be observed that the applied load on both sides of the specimen tends to open the crack, and the displacement distribution reflects this behavior. To quantify the crack opening displacement (CTOD), symmetrical points located on either side of the crack flanks, just behind the crack tip, are selected. Figure 6b displays the displacement loops obtained for symmetrical points positioned at 0.1, 0.2, 0.3, and 0.4 mm behind the crack tip. As expected, the amplitude of the CTOD increases with the distance from the crack tip. From these curves, the plastic component of the CTOD was extracted following the methodology described in Section 2.1. Moreover, the change in slope observed at low load levels is a characteristic feature of the crack closure mechanism acting on the crack flanks.

As shown, the CTOD curves in Figure 6b display a trend comparable to that in Figure 1. The beginning of the curve indicates the progressive separation of the crack flanks at the measurement point as the load is applied, and it becomes linear once the crack is fully open. The slope of this linear segment characterizes the elastic response of the material. By extrapolating this elastic slope to the loading portion of the curve, the plastic component of the CTOD can be determined, as shown in Figure 1. As verified in previous studies [13,20,21], the plastic CTOD values obtained from DIC analysis are dependent on the measurement position, reaching their maximum approximately 100–150 µm behind the crack tip. The methodology consists of obtaining CTOD measurements along the crack surfaces, analyzing the corresponding plastic CTOD values, and recording the maximum value for each crack length.

**Figure 7:**
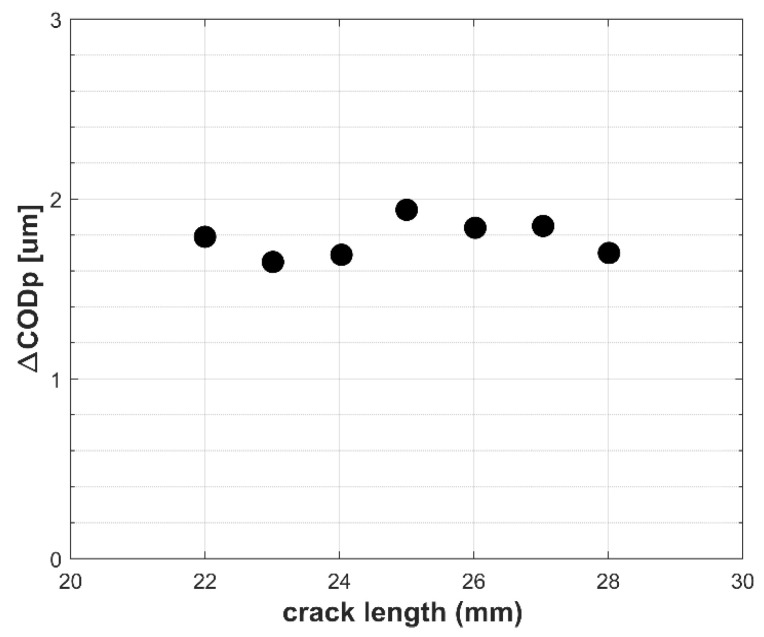
Plastic CTOD measurements obtained during the constant-ΔK fatigue test.

Table 3 shows the values of plastic CTOD obtained for crack lengths ranging from 22 to 28 mm, which were subsequently plotted in Figure 7. The results indicate that the plastic CTOD remains approximately constant across all examined crack lengths, reflecting the constant ΔK maintained during the fatigue test. Small deviations are attributed to changes in material stiffness with crack growth, which require continuous load adjustments to ensure constant ΔK.

**Figure 8:**
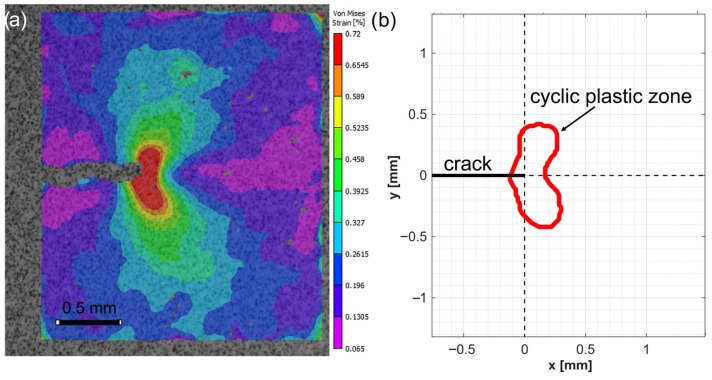
(**a**) von Mises strain field obtained via DIC at a crack length of 22 mm. (**b**) Corresponding geometry of the cyclic plastic zone.

In the next step of the analysis, the extension of the cyclic plastic zone is determined based on the experimental data. For this purpose, the unloading path at each crack length was examined using DIC analysis, as the cyclic plastic zone originates at the crack tip while the load returns to its minimum value. As strain is the variable of interest, the image captured at maximum load was set as the reference to evaluate the strain evolution during unloading. Therefore, the region that characterizes the cyclic plastic zone can be determined. Figure 8a shows the results of this procedure for a crack length of 22 mm, where the von Mises strain was calculated from the DIC data, offering a more comprehensive representation of the strain state. By applying the methodology described in Section 2.2, the shape of the cyclic plastic zone was determined and is shown in Figure 8b. The examined zone exhibits a butterfly-shaped distribution pattern, where the highest strain values are concentrated in front of the crack tip. This distribution is consistent with the development of a pronounced strain gradient in that region, clearly reflecting the localized material deformation induced and progressively intensified by cyclic loading conditions.

**Figure 9:**
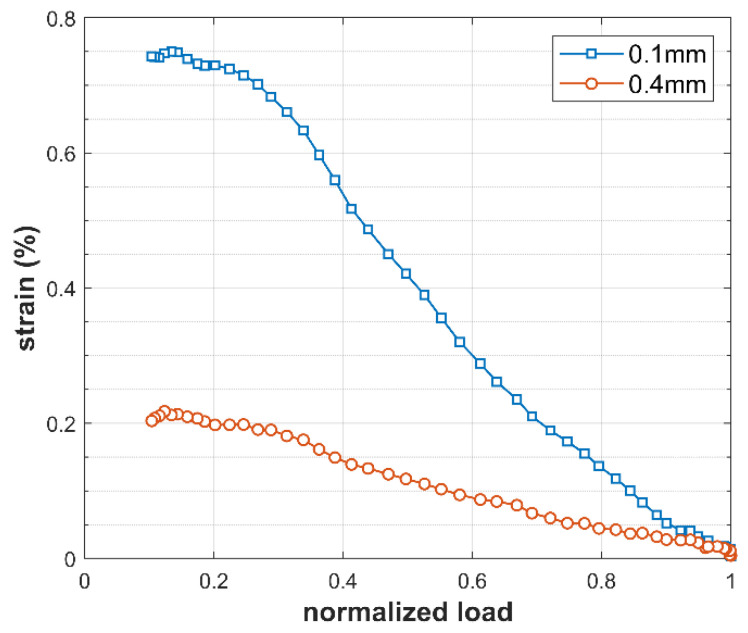
Strain evolution during unloading at two points located 0.1 mm and 0.4 mm ahead of the crack tip.

In addition, Figure 9 shows the evolution of strain along the unloading path for two points located 0.1 mm and 0.4 mm ahead of the crack tip. As observed in the strain contour in Figure 8b, the point at 0.1 mm lies within the estimated cyclic plastic zone, whereas the point at 0.4 mm is located outside it. The difference in strain values between these two points is significant, showing the high strain gradient that develops in this region. Furthermore, it is noteworthy to observe the effect of the crack closure mechanism at low loads, protecting the crack tip from experiencing higher strain values during unloading.

**Figure 10:**
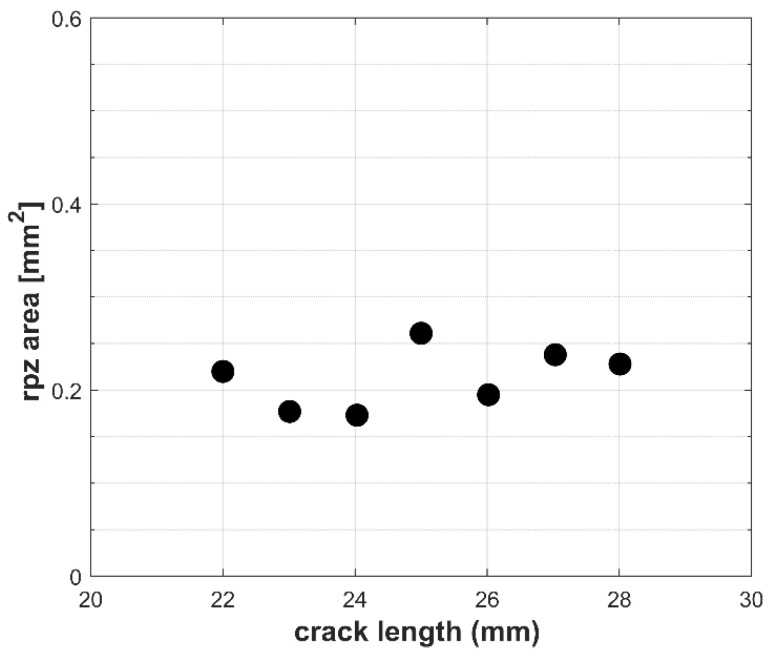
Evolution of the cyclic plastic zone area throughout the constant-ΔK fatigue test.

Finally, Table 4 shows the size of the cyclic plastic zone (rpz, from reverse plastic zone) obtained for crack lengths ranging from 22 to 28 mm, which were subsequently plotted in Figure 10. The figure illustrates the variation in cyclic plastic zone area for all measured crack lengths, expressed in terms of the area encompassed by the boundary points. It can be observed that, similar to the plastic CTOD measurements, the area of the cyclic plastic zone remains nearly constant throughout crack growth. This behavior aligns with the application of a constant ΔK during the fatigue crack propagation, indicating a nearly constant cyclic plastic deformation zone as the crack propagates.

## 4. Discussion

This study investigates two nonlinear parameters: the plastic crack-tip opening displacement (ΔCTOD_p_) and the cyclic plastic zone size. Both parameters quantify crack-tip plasticity in different regions around the crack tip. While the plastic CTOD is determined from the crack-tip opening response measured behind the crack tip, reflecting the permanent deformation accumulated during loading, the cyclic plastic zone size is derived from the plastic deformation field that develops ahead of the crack tip, representing the region subjected to cyclic yielding during crack propagation. The objective of this study is to perform a fatigue crack propagation test under constant-ΔK conditions and to evaluate both parameters. Since constant-ΔK conditions are maintained throughout the test, the driving forces governing crack propagation should also remain constant. Therefore, conducting a constant-ΔK test enables a more controlled assessment of crack growth behavior and fracture parameters, as it isolates the effect of ΔK from the influence of varying load conditions. This approach differs from a constant-ΔP test, in which the stress intensity factor (K) increases as the crack extends.

At first, the crack-tip opening response is evaluated trough the CTOD parameter, which captures the combined contributions of elastic and plastic deformation at the crack tip. The initial segment of the CTOD curve, corresponding to the gradual unzipping of the crack faces due to the crack closure mechanism acting on the crack surfaces, was successfully captured by the methodology, as shown in Figure 6b. This behavior is indicative of the crack-opening process, allowing the identification of the fully open crack state and the onset of plasticity ahead of the crack tip. This characterization further supports the reliability of the plastic CTOD as a measure of crack-tip deformation.

On the other hand, the study of the cyclic plastic zone size is also motivated by its direct relationship with crack-tip plasticity. From Figure 9, it was observed that the premature contact of the crack flanks shields the crack from the full influence of the applied elastic ΔK. Therefore, the effects of plasticity-induced crack closure are inherently included in the reversed plastic zone size.

For both analyses, the plastic CTOD was approximately 1.78 μm ± 0.10 μm, and the plastic zone size was about 0.213 μm ± 0.033 μm, shown in Figure 7 and Figure 10, respectively. The minor variations observed in both parameters are likely due to progressive changes in material stiffness as the crack propagates, requiring continuous load adjustments to maintain the constant ΔK. However, they exert no significant influence on the overall assessment of the crack-tip plasticity parameters.

The da/dN plot shown in Figure 2 indicates that the crack propagated under nearly constant values of ΔK. The results obtained from the plastic CTOD (ΔCTOD_p_) and the cyclic plastic zone size (rpz), also display nearly constant values throughout the test. Therefore, it can be concluded that the quasi-constancy of these nonlinear parameters under constant-ΔK conditions confirms that the crack-tip deformation reaches a quasi-steady condition for the loading conditions tested here. This consistent behavior highlights their potential as key parameters for describing fatigue crack propagation, as they effectively quantify crack-tip plasticity and inherently account for critical plasticity-induced phenomena such as crack closure, crack-tip blunting, and the influence of residual stresses.

In fact, fatigue cracks propagate through regions of the material that were previously deformed by the plastic zone generated ahead of the crack tip. In ductile materials, fatigue crack propagation is primarily governed by crack-tip plasticity. Therefore, nonlinear parameters such as ΔCTOD_p_ and the cyclic plastic zone size provide a direct physical basis for describing and understanding the mechanisms driving fatigue crack growth. Future work should focus on confirming these observations across different materials and more complex loading scenarios where plasticity effects are more pronounced.

## 5. Conclusions

This experimental study demonstrated that nonlinear parameters, such as the plastic crack-tip opening displacement (CTOD_p_) and the cyclic plastic zone size, can be used as potential driving-force parameters for characterizing fatigue crack growth. From the experimental results, it can be observed that a direct relationship exists between both parameters and the crack growth rate (da/dN). The quasi-constancy of these parameters under constant-ΔK conditions confirms that the crack-tip deformation reaches a quasi-steady state for the tested loading conditions.

These findings are in agreement with the observation that the force or forces actually governing crack propagation should also remain nearly constant during the constant-ΔK tests. The analysis of both nonlinear parameters has proven highly useful, as they serve as effective indicators of crack-tip plasticity phenomena, providing a direct measure of local deformation. Moreover, these parameters offer insights that go beyond conventional linear–elastic fracture mechanics parameters, making them particularly valuable for assessing crack-tip behavior in materials exhibiting significant plasticity.

Furthermore, the use of high-magnification 3D-DIC analysis significantly enhances the reliability of the results. This technique accurately quantifies the surface behavior of the material, including depressions caused by high strain levels near the crack tip. It is well-established that large plastic strains at a crack tip can cause surface roughening, intrusions/extrusions, and depressions; accurately measuring these effects is crucial for understanding crack-tip mechanics. Consequently, such critical features would remain undetectable using a conventional 2D-DIC analysis.

## Figures and Tables

**Table 3:** Values of ΔCOD_p_ obtained from the analysis.

Crack Length (mm)	ΔCOD_p_ (µm)
22	1.79
23	1.65
24	1.69
25	1.94
26	1.84
27	1.85
28	1.70

**Table 4:** Values of cyclic plastic zone (rpz) obtained from the analysis.

Crack Length (mm)	rpz (mm^2^)
22	0.220
23	0.177
24	0.173
25	0.261
26	0.195
27	0.238
28	0.228

## Data Availability

The original contributions presented in this study are included in the article. Further inquiries can be directed to the corresponding author.

## Abbreviations

The following abbreviations are used in this manuscript:

CJP	Christopher–James–Patterson
CTOD	Crack-Tip Opening Displacement
CTOD_p_	Plastic Crack-Tip Opening Displacement
DIC	Digital Image Correlation
DCT	Disk-shaped Compact Tension
*E*	Young’s Modulus
K	Stress Intensity Factor
*h*′	Cyclic Strain Hardening Exponent
*H*′	Cyclic Strength Coefficient
LEFM	Linear Elastic Fracture Mechanics
R	Load Ratio
rpz	Reverse Plastic Zone
*V*	Displacement in *y*-Direction
ΔK	Stress Intensity Factor Range
Δ*ε*	Strain Range
Δ*σ*	Stress Range
*ε*	Strain
*ε*_y_, *ε*_cy_	Yield Strain, Cyclic Yield Strain
*σ*	Stress
*σ*_y_, *σ*_cy_	Yield Stress, Cyclic Yield Stress
